# Emergency Medicine Physicians Would Prefer Using Cannabis Over Opioids for First-Line Treatment of a Medical Condition if Provided With Medical Evidence: A National Survey

**DOI:** 10.7759/cureus.19641

**Published:** 2021-11-16

**Authors:** Kevin M Takakuwa, Raquel M Schears

**Affiliations:** 1 Emergency Medicine, Society of Cannabis Clinicians, Sebastopol, USA; 2 College of Medicine, University of Central Florida, Orlando, USA

**Keywords:** emergency medicine, treatment preference, national survey, opioid epidemic, emergency department, medical cannabis

## Abstract

Objective: To determine emergency medicine (EM) physicians’ preferences for using medical cannabis versus opioids if medical cannabis was legalized.

Methods: We surveyed US physicians at the largest national EM conference (American College of Emergency Physicians’ Annual Conference) held in San Diego, CA from October 1 to 4, 2018. Of the thousands of conference participants approached, 539 US physicians completed the anonymous written survey, which represented 15.2% of the US physicians attending the conference.

Results: The mean age of the participants was 39.6 ± 10.9 years, men composed 57.5% of the participants, and whites made up 72.8% of the respondents. Participants practicing in medically legal (54.8%) and medically plus adult-use legal cannabis states (23.1%) totaled 77.9%. A majority (70.7%) of the participants believed that cannabis has medical value. EM physicians preferred cannabis over opioids as a first-line treatment addressing a medical condition provided that medical studies found that cannabis was equally effective (p < 0.001, *X*^2 ^= 36.8 [95% CI 2, 415]), and overwhelmingly preferred cannabis over opioids if it were more effective (p < 0.001, *X*^2 ^= 90.8 [95% CI 2, 415]). Physicians appeared to prefer opioids over cannabis if medical studies found that cannabis was less effective though it was not significant (p > 0.05). Subgroup analyses showed that belief in the medical value of cannabis significantly increased the odds ratio of choosing cannabis over opioids if cannabis was equally or more effective than opioids.

Conclusion: Our study shows that EM physicians believe cannabis has medical value and would prefer using cannabis over opioids if provided with equivalent findings. We believe our findings reflect EM physicians’ experience of the opioid epidemic and suggest the need for further study of this potential therapeutic.

## Introduction

Opioid-related deaths claimed 446,032 lives in the US between 1999 and 2018 [1] and emergency medicine (EM) physicians are on the frontlines of care from resuscitating overdoses to managing acute pain while counterbalancing opioid-seeking behavior. Because EM physicians are acutely aware that administering opioids in the emergency department and writing outpatient opioid prescriptions--frequently requested by patients--may contribute to opioid misuse and dependence, many contemplate alternative treatments. One such alternative is medical cannabis, which has been proposed as an alternative or adjunct to opioids for pain diminution [2,3], particularly chronic pain [4-9]. Since medical cannabis is currently federally illegal and there has never been a study to determine if EM physicians would even consider its use in an acute setting, our objective was to assess EM physicians’ preferences for using medical cannabis compared to opioids in an EM setting were it to become legalized.

## Materials and methods

This was a survey questionnaire of US physicians who attended the largest national EM conference (American College of Emergency Physicians [ACEP] Annual Conference) held in San Diego, CA from October 1 to 4, 2018. There were 3,536 US physicians in attendance out of 7,479 registered conference attendees. The inclusion criteria for this study were self-identification as being a US physician and willingness to complete the survey. Therefore, all who did not meet these criteria were excluded. To obtain our sample, a single physician surveyor (KMT) was positioned just outside of conference registration at a coffee stand and conducted a convenience survey of conference participants for the first three days of the conference from 09:00 until 14:00. He asked, “Hello, are you a US physician?” If the response was affirmative, he followed up with, “Would you be willing to fill out a one-minute anonymous survey for the ACEP ethics committee?”

The authors of this study wrote a 16-question survey that included nine demographic questions (age, race, gender, medical degree [Doctorate of Medicine {MD}, Doctorate of Osteopathic Medicine {DO}, other], primary medical specialty, number of years of residency completed, number of years of practice after residency, medical practice setting, and primary state of practice), three questions comparing cannabis with opioids with assumptions on efficacy, and a question “Do you believe cannabis has any medical value?” (see Appendix). There were additional questions about what indications EM physicians would consider using cannabis as well as whether they would use it in pediatric or pregnant patients, the results of which were published in another paper [10].

Thousands of conference attendees were approached over the first three days of the conference, 549 completed the written survey and 10 were excluded (five from non-US practicing physicians, three from non-physicians, and two for illegible and incomprehensible survey responses). This left 539 US physicians who completed the anonymous written survey, which represented 15.2% of the US physicians attending the conference. The survey was a quality improvement initiative of the ACEP ethics committee and Institutional Review Board approval was obtained from the University of Central Florida (00001165).

Frequency and percentage, mean, and standard deviations were reported for survey responses. To compare demographic variables, age, gender, race (white versus non-white), practice setting (academic versus non-academic), medical specialty (EM versus internal and family medicine), type of medical degree (MD versus DO), years of practice (nine years or less versus 10 years or more), cannabis legality in state of practice (legal medically and/or adult-use versus not legal), and belief that cannabis has medical value, to three cannabis versus opioids scenarios, chi-square tests for independence and Fisher’s exact test were performed. Continuous variables such as age were dichotomized (age: <40 and >40 years). All analyses were performed using SPSS statistical software (version 25, IBM Corp., Armonk, NY). A p-value <0.05 was considered statistically significant.

## Results

The mean age of the participants was 39.6 ± 10.9 years. Men composed 57.5% of the participants. Whites made up 72.8%. Respondents practicing in medically legal (54.8%) and medically plus adult-use legal cannabis states (23.1%) totaled 77.9%. All US states plus the District of Columbia and Puerto Rico were represented in the survey except for four states (Table 1). A majority (70.7%) of participants believed that cannabis has medical value.

**Table 1:** Respondents by state legality as of October 2018. * Percentages may not total 100% due to missing data or rounding. ^1^ Illinois passed adult use in 2019. ^2^ Michigan passed adult use in November 2018. ^3^ Missouri passed medical use in November 2018. ^4^ Utah passed adult use in November 2018.

Legality	States of practice	N	%	Total N (%)
Both (medical and adult)	California	61	11.5*	
	Massachusetts	14	2.7	
	Oregon	13	2.5	
	Washington	11	2.1	
	Colorado	6	1.1	
	Alaska	5	0.9	
	District of Columbia	5	0.9	
	Nevada	5	0.9	
	Vermont	2	0.4	122 (23.1)
Medical	New York	53	10.0	
	Florida	33	6.2	
	Illinois^1^	33	6.2	
	Michigan^2^	30	5.7	
	Ohio	28	5.3	
	Pennsylvania	20	3.8	
	New Jersey	17	3.2	
	Minnesota	10	1.9	
	Connecticut	9	1.7	
	New Mexico	9	1.7	
	Puerto Rico	7	1.3	
	Maryland	6	1.1	
	Arizona	5	0.9	
	Arkansas	5	0.9	
	Maine	5	0.9	
	Delaware	4	0.8	
	Louisiana	4	0.8	
	Oklahoma	4	0.8	
	Hawaii	3	0.6	
	Montana	2	0.4	
	New Hampshire	2	0.4	
	Rhode Island	1	0.2	
	North Dakota, West Virginia	0	0	290 (54.8)
Not legal	Texas	25	4.7	
	Georgia	16	3.0	
	Tennessee	11	2.1	
	North Carolina	10	1.9	
	South Carolina	10	1.9	
	Indiana	8	1.5	
	Virginia	6	1.1	
	Wisconsin	6	1.1	
	Iowa	4	0.8	
	Missouri^3^	4	0.8	
	Kansas	3	0.6	
	Kentucky	3	0.6	
	Mississippi	3	0.6	
	Utah^4^	3	0.6	
	Alabama	2	0.4	
	Nebraska	2	0.4	
	Wyoming	1	0.2	
	Idaho, South Dakota	0	0	117 (22.1)

Provided with the premise that cannabis was to become federally legal, administrable in different formats besides smoking (e.g., pills, sublingual, and intravenous), and available for use in an EM setting, EM physicians preferred cannabis over opioids as a first-line treatment for treating a medical condition provided that medical studies found that cannabis was equally effective (p < 0.001, *X*^2^ = 36.8 [95% CI 2, 415]), and overwhelmingly preferred cannabis over opioids if it were more effective (p < 0.001, *X*^2^ = 90.8 [95% CI 2, 415]). Physicians seemed to prefer opioids over cannabis if medical studies found that cannabis was less effective though it was not significant (p > 0.05). Subgroup analyses showed that belief in the medical value of cannabis significantly increased the odds ratio of choosing cannabis over opioids if cannabis was found equally or more effective than opioids but did not reveal any other significant findings for the remaining eight demographic variables collected except that whites preferred opioids over cannabis if cannabis was shown to be less effective (Table 2).

**Table 2:** Responses to comparisons of opioids versus cannabis assuming medical study evidence for treating a medical condition. * Percentages may not total 100% due to missing data or rounding. ^1^ Comparing cannabis to opioids for belief in medical value of cannabis, p < 0.001 (OR = 23.75 [95% CI 5.20, 108.42]). ^2^ Comparing cannabis to opioids for belief in medical value of cannabis, p < 0.001 (OR = 32.90 [95% CI 7.41, 146.00]). ^3^ Comparing cannabis to opioids for whites, p < 0.05 (OR = 1.97 [95% CI 1.11, 3.50]).

Preferences for first-line treatments if:	Cannabis		Opioids		Don't know/no preference	
	N	%	N	%	N	%
Cannabis was as equally effective as opioids^1^	282	(52.3)	26	(4.8)	231	(42.9)
Cannabis was more effective than opioids^2^	429	(79.6)	11	(2.0)	99	(18.4)
Cannabis was less effective than opioids^3^	66	(12.2)	267	(49.5)	206	(38.2)

## Discussion

The finding of 70.7% of EM physicians who believed cannabis has medical value was consistent with a similar study we published in the previous year reporting 68.3% of EM physicians believed in its medical value [11]. While there was a large percentage of physicians who chose “don’t know/no preference,” which we believe suggests ambivalence or lack of knowledge [12,13] about a substance classified as federally illegal [14], we were surprised that physicians preferred medical cannabis (a Schedule I drug) in an EM setting compared to opioids (a Schedule II drug, a less restrictive drug category) when it was proposed as equally and especially more effective, particularly for those who hold the belief that cannabis has medical value.

We did not expect EM physicians would be willing to use cannabis in an EM setting at this point in time given its decades of stigma as a dangerous drug [15-17]. While cannabis has previously been used medicinally in the US [18], its subsequent federal scheduling has prevented the type of rigorous research needed to prove or disprove its efficacy [6]. In a comprehensive review of medical cannabis, the National Academies of Sciences concluded that cannabis is useful for treating adult chronic pain [6]. With growing public support for its medicinal uses [19], other conditions including nausea, anxiety, and depression are being targeted as treatment potentials, which might be useful in an EM setting.

With few randomized controlled clinical trials and a paucity of clinical research delving into the extensive endocannabinoid system [6], physicians have had to formulate opinions on cannabis without comprehensive medical literature. Since cannabis has not been used in hospital or emergency care settings, and there are no current indications for acute use, we were somewhat surprised at the theoretical willingness to incorporate this treatment into EM. Perhaps this is consistent with working in a dynamic environment that may require flexible solutions but we believe more likely suggests the strong desire to have treatment options for pain besides opioids.

EM physicians are acutely aware of the impact of the opioid epidemic in their daily practice. In a report from the Centers for Disease Control, 15.7 per 10,000 ED visits were suspected of opioid overdoses [20]. They are equally aware of the almost impossible balancing act of treating pain without initiating or exacerbating opioid misuse or dependence. One study reported that EM physicians prescribed opioids in 15-20% of ED visits [21]. Another found that EM physicians were found to have initiated 11.7-17.4% of all opioid prescriptions in an administrative claims database of opioid naïve patients [22]. They may also be aware of the study showing that the addition of cannabis to opioids diminished the use of opioid medication while maintaining analgesia [23], that the safety profile of cannabis is low compared to tobacco, alcohol, and other illicit drugs including opioids [24], and that increasing studies are reporting medical benefits of cannabis [6].

We do caution that an important caveat to our study results were the assumptions that cannabis was federally legal, could be administered in various formats other than smoking, and that medical studies showed the efficacy of cannabis relative to opioids. Certainly, our current state of practice is far from meeting these assumptions from both a political and research standpoint. Despite this, the importance of this study is that EM physicians are willing to consider alternative options to opioids and might support cannabis usage in their practice. Whether our study has external validity is yet to be determined. Hopefully, our study will stimulate further research into areas in which cannabis may be useful for patients in an acute setting.

## Conclusions

We believe our results reflect EM physicians’ desire to have alternative treatment options to the known potentially addictive effects of opioids, understanding the gravity of the opioid epidemic, and the biases of dealing with opioid-related issues in EM. Considering that the “don’t know/no preference” percentage decreased in favor of cannabis when cannabis was posed as more effective than opioids, our results suggest that EM physicians appear willing to use medical cannabis in an EM setting should they be provided with sufficient medical evidence. Further study of this potential therapeutic is warranted.

## Figures and Tables

**Figure 1:**
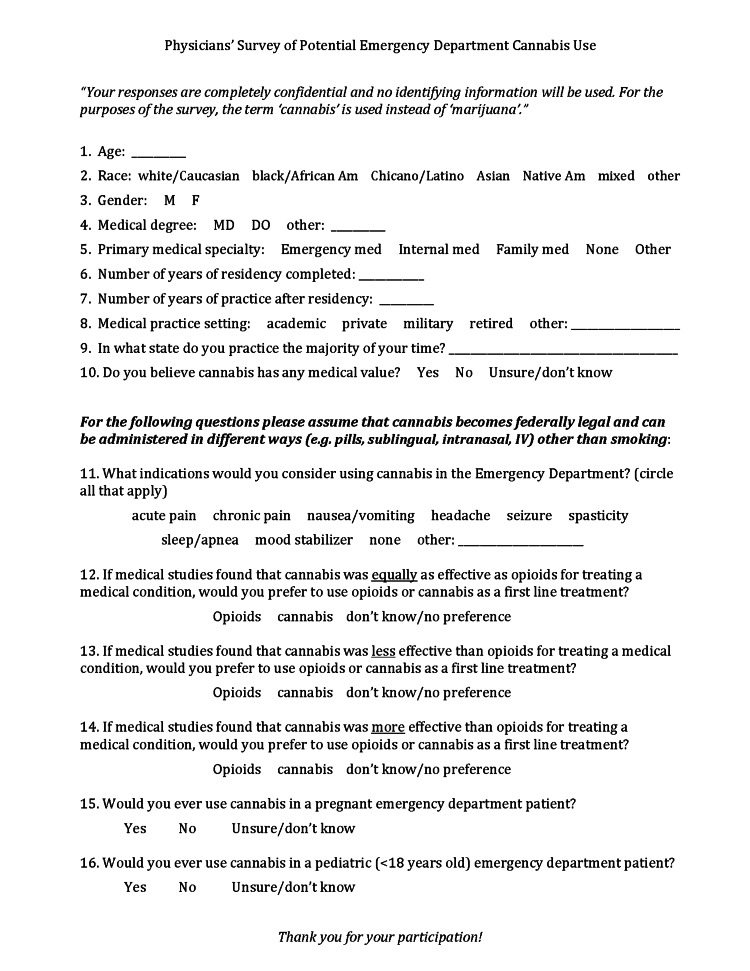
Survey questionnaire.

## References

[1] Wilson N, Kariisa M, Seth P, Smith H 4th, Davis NL (2020). Drug and opioid-involved overdose deaths - United States, 2017-2018. MMWR Morb Mortal Wkly Rep.

[2] Roberts JD, Gennings C, Shih M (2006). Synergistic affective analgesic interaction between delta-9-tetrahydrocannabinol and morphine. Eur J Pharmacol.

[3] Takakuwa KM, Sulak D (2020). A survey on the effect that medical cannabis has on prescription opioid medication usage for the treatment of chronic pain at three medical cannabis practice sites. Cureus.

[4] Aggarwal SK (2013). Cannabinergic pain medicine: a concise clinical primer and survey of randomized-controlled trial results. Clin J Pain.

[5] Haroutounian S, Ratz Y, Ginosar Y, Furmanov K, Saifi F, Meidan R, Davidson E (2016). The effect of medicinal cannabis on pain and quality-of-life outcomes in chronic pain: a prospective open-label study. Clin J Pain.

[6] National Academies of Sciences, Engineering Engineering, and Medicine (2017). The Health Effects of Cannabis and Cannabinoids: The Current State of Evidence and Recommendations for Research.

[7] Busse JW, Wang L, Kamaleldin M (2018). Opioids for chronic noncancer pain: a systematic review and meta-analysis. JAMA.

[8] Mücke M, Phillips T, Radbruch L, Petzke F, Häuser W (2018). Cannabis-based medicines for chronic neuropathic pain in adults. Cochrane Database Syst Rev.

[9] Takakuwa KM, Hergenrather JY, Shofer FS, Schears RM (2020). The impact of medical cannabis on intermittent and chronic opioid users with back pain: how cannabis diminished prescription opioid usage. Cannabis Cannabinoid Res.

[10] Takakuwa KM, Schears RM (2021). Indications and preference considerations for using medical cannabis in an emergency department: a national survey. Am J Emerg Med.

[11] Takakuwa KM, Shofer FS, Schears RM (2020). The practical knowledge, experience and beliefs of US emergency medicine physicians regarding medical cannabis: a national survey. Am J Emerg Med.

[12] Evanoff AB, Quan T, Dufault C, Awad M, Bierut LJ (2017). Physicians-in-training are not prepared to prescribe medical marijuana. Drug Alcohol Depend.

[13] Takakuwa KM, Mistretta A, Pazdernik VK, Sulak D (2021). Education, knowledge, and practice characteristics of cannabis physicians: a survey of the Society of Cannabis Clinicians. Cannabis Cannabinoid Res.

[14] (2021). US Drug Enforcement Agency (DEA). Office of Diversion Control. Controlled substance schedules and list of controlled substances. https://www.deadiversion.usdoj.gov/schedules/.

[15] Bonnie RJ, Whitebread CH II (1974). The Marihuana Conviction: A History of Marihuana Prohibition in the United States. Charlottesville: University Press of Virginia.

[16] Schlosser E (2021). The Atlantic. Reefer madness. The Atlantic.

[17] Takakuwa KM (2020). A history of the Society of Cannabis Clinicians and its contributions and impact on the US medical cannabis movement. Int J Drug Policy.

[18] (2021). The Pharmacopeia of the United States of America. By authority of the National Medical Convention. https://babel.hathitrust.org/cgi/pt?id=uc1.b4330104;view=1up;seq=7.

[19] (2021). Pew Research Center. 6 facts about Americans and marijuana. https://www.pewresearch.org/fact-tank/2021/04/26/facts-about-marijuana/.

[20] Vivolo-Kantor AM, Seth P, Gladden RM, Mattson CL, Baldwin GT, Kite-Powell A, Coletta MA (2018). Vital signs: trends in emergency department visits for suspected opioid overdoses - United States, July 2016-September 2017. MMWR Morb Mortal Wkly Rep.

[21] Ali MM, Cutler E, Mutter R, Henke RM, Mazer-Amirshahi M, Pines JM, Cummings N (2019). Opioid prescribing rates from the emergency department: down but not out. Drug Alcohol Depend.

[22] Jeffery MM, Hooten WM, Hess EP (2018). Opioid prescribing for opioid-naive patients in emergency departments and other settings: characteristics of prescriptions and association with long-term use. Ann Emerg Med.

[23] Nielsen S, Sabioni P, Trigo JM (2017). Opioid-sparing effect of cannabinoids: a systematic review and meta-analysis. Neuropsychopharmacology.

[24] Lachenmeier DW, Rehm J (2015). Comparative risk assessment of alcohol, tobacco, cannabis and other illicit drugs using the margin of exposure approach. Sci Rep.

